# Mothers’ experiences and satisfactions with health extension program in Jimma zone, Ethiopia: a cross sectional study

**DOI:** 10.1186/1472-6963-13-74

**Published:** 2013-02-21

**Authors:** Zewdie Birhanu, Ameyu Godesso, Yohannes Kebede, Mulusew Gerbaba

**Affiliations:** 1 Department of Health Education and Behavioral Sciences, College of Public Health and Medical Sciences, Jimma University, Jimma, Ethiopia; 2 Department of Sociology and Social Work, College of Social Sciences and Law, Jimma University, Jimma, Ethiopia; 3 Department of Population and Family Health, College of Public Health and Medical Sciences, Jimma University, Jimma, Ethiopia

**Keywords:** HEWs, Experiences, Satisfactions, HEP, Ethiopia

## Abstract

**Background:**

Although mothers are the fundamental unit of interventions in Health Extension Program in Ethiopia, their experiences and satisfactions with the service remain unstudied. Therefore, this study was aimed to assess mothers’ experiences and satisfaction with health extension service.

**Methods:**

A community based cross sectional study was conducted in Jimma Zone, Oromiya, Ethiopia. Three hundred Seventy-nine mothers were participated in the study. The study was conducted in four randomly selected rural villages. Systematic sampling technique was used to identify respondents. As part of the data collection process, four focus group discussions were conducted with mothers. SPSS 17.0 and ATLASti.4.1. Softwares were used for data analysis.

**Results:**

One hundred Sixty nine (51.7%) of the respondents had an experience of interactions with health extension workers during one year prior to the survey, while 271 (71.5%) of them reported that they received visits from health extension workers during the same period. 298 (78.6%) of the respondents received information at least on one of the Health extension packages. In fact, they had better exposure to personal hygiene and environmental sanitation packages. Even though health extension program is being run by female workers alone, it was believed that the involvement of men is vital to the success of the program. Mothers thought that men are more courageous and professionally competent to deal with complex matters. They also tended to criticize health extension program for lacking curative services and health extension workers are less competent. The greater emphasis laid on outreach services was not supported. 286 (75.5%) of the respondents rated their relationship with health extension workers as positive. Similarly, higher satisfaction was reported though the program has problems. Age, perceived skill to diagnose community problems, perceived respect, involvement of husband and being recognized as a model family were significantly predicted satisfactions with health extension services.

**Conclusions:**

Most mothers had good relationship, were satisfied with and had positive attitude towards health extension program though the program was criticized for not including curative services and the less attention given to static services at health post. Stakeholders are required to reconsider these issues.

## Background

Despite the encouraging trends, Ethiopia still has several poor key health outcome indicators relative to other low-income countries, even within sub-Saharan Africa. This is largely attributed to the prevalence of preventable infectious diseases, ailments and nutritional deficiencies and poor access to health services [1-4]. To address the health needs of the population, the government of Ethiopia has launched a comprehensive Health Sector Development Plans (HSDP) in 2003/04 [3,4]. HSDP is a 20 year plan divided into 3–5 year rolling plans in four consecutive phases. It was developed in response to the prevailing and newly emerging health problems and in recognition of weaknesses in the existing health delivery system [3,4]. As part of the HSDP, the government has introduced an innovative health program called Health Extension Program (HEP) in 2002/03 [4,5].

HEP is an innovative community based health service delivery program targeting households. The program consist of a package of basic and essential promotive, preventive and few selected high impact curative health services. It is designed to improve the health status of families, with their full participation, using local technologies and the community’s skills and wisdom [5]. The philosophy of HEP is that if the right knowledge and skill is transferred to households, they can take responsibility for producing and maintaining their own health. The program was designed with the premises of accelerating the country’s progress in meeting health related Millennium Development Goals [4,5]. HEP is composed of four main themes: Disease Prevention and Control, Family Health, Hygiene and Environmental Sanitation and Health Education and Communication.

These four themes consist of about sixteen health packages which mainly deal with promotive and preventive health services [5]. The program is implemented by new health carders called Health Extension Workers (HEWs) who were trained solely for the implementation of the program. Basically, all HEWs are women who completed grade ten and received technical and vocational training for one year [5]. By the end of 2010, a total of 33,819 HEWs were trained and deployed in each rural village throughout Ethiopia to serve the community [3]. Two HEWs were assigned to each Ganda throughout the country. Ganda is the smallest administrative unit in Oromiya, Ethiopia. Each Ganda has a Health Post (HP) which serves as the operational center for two HEWs. These new cadres were selected from the communities in which they reside in order to ensure acceptance by community members. They are the first point of contact of the community within the health care delivery system [4,5].

The main task of HEWs is increasing the knowledge and skill of the community members and households to deal with communicable diseases and be able to access to health services with especial attention to maternal and child health. The fact that maternal and child health package is the milestone in the program; mothers or women are the fundamental unit of interventions for HEP [5]. HEWs are required to spend 75% of their time conducting house –to- house activities to teach and help households and community members to adopt healthy behaviors. Besides providing health education on family health, environmental sanitations and common communicable diseases (TB, malaria and HIV/AIDS), HEWs supervise Directly Observable Treatment-Short Course (DOTS) for TB and antiretroviral treatment for HIV/AIDS; conduct rapid diagnostic tests for malaria and administer artemether/lumefantrine; provide family planning and immunization services; attend uncomplicated childbirth and refer patients to nearby health centers. However, HEWs are not allowed to administer antibiotics [5,6]. HEP educational approach is based on training model families that have acceptance and credibility by the community, as early adopters of desirable health practices to become role models in line with heath extension packages. It was expected that all households would be graduated as models within three years of the implementation of the program [5,6]. However, in 2010 only 26% of the households received title of model family [3].

Evidence has shown that at the heart of any health service delivery system is a positive relationship between clients and providers and in fact, it is likely to remain true for the foreseeable future [7]. More importantly, such indispensable aspect of care is clearly fundamental in the future of care where health promotion and health education activities are more important and the primary units of interventions are households. Although mothers are the primary target group for HEP, their experiences with the program remains unstudied in Ethiopia. This is evidenced by the fact that the majority of scientific inquiries related to HEP were primarily focused on implementation status of the program [8-10], efficiency of HEWs [11], working conditions and experiences of HEWs [12,13], access to information and continuing education [14], and effects of the program [15]. Only one study, as we were able to identify from published evidences, described the initial community experiences on HEP [16]. Therefore, it is timely and appropriate to assess mothers’ experiences with HEP as they are the fundamental unit of interventions for most of the HEP packages. Hence, this study was primarily intended to assess mothers’ experiences and satisfactions with HEP.

## Methods

### Study setting

Community based cross sectional study was conducted in four randomly selected districts in Jimma Zone, Oromiya National Regional State (Ethiopia) over a period of two months (November- December 2011). The zone comprises of 17 districts with a total population of 2,495,795 of whom females account for 49.7%. Each district has at least one health center with five satellite HPs. In addition, each Ganda has one health post that services as operational center for HEWs. About 94.3% of the populations of the zone are rural dwellers [17].

### Population

The study was conducted on women of reproductive age group (15–49 years) who reside in the rural Gandas of Jimma Zone. Women were preferred for this study due to the fact that most of the services rendered by HEWs deal with mothers at household level. Respondents were included in the study if they have lived in the selected Ganda at least for six months. However, urban Gandas were not included in the study because of the recent initiation of HEP at urban level which makes it premature to measure the study variables in such context.

### Sample size

The sample size was calculated using single population proportion formula (n = (Z 1-α/2)^2^ p (1-p)/ d^2^) with thefollowing assumptions: expected proportion (p) of the study participants who were satisfied with HEP (50%), marginal error (d) 5% and confidence interval of 95%. A proportion of 50% was preferred due to lack of similar studies. This yields a sample size of 384 respondents. Considering 5% non-response rate, the final sample size was determined to be 403. For the qualitative part of the study, four FGDs were conducted with mothers (one FGD per Ganda). In each FGD, 6–10 participants were participated.

### Sampling technique

In Jimma Zone, four districts were selected randomly. Then, one functional HP was randomly identified from each district. Respondents were recruited from the Ganda which is being served by the selected HPs. The total number of women in the reproductive age group was indentified in each Ganda for proportional sample size allocation. Finally, systematic sampling technique was employed to identify the respondents. The sampling unit was a household and the sampling interval was determined based on the number of households in the selected Gandas with the assumption that one eligible respondent could be available in each household. In case two women were available within one household, one woman was selected based on exposure to HEP. FGD participants were women, aged 25–45 years, selected purposively considering the level of exposure and contact with HEP at their respective Gandas. All of them did not attend formal education.

### Measurements

Instruments were developed through thorough review of documents, guidelines and manuals related to HEP, and relevant literatures [5,10,13,15,16,18]. The questionnaire consisted of three main parts: the first part was composed of socio-demographic information of the respondents. The second part consisted of items related to exposure to and experiences with health HEP and was presented in ‘yes-no’ format. The items in the third category were intended to measure respondents’ orientations and perceptions on HEP. Initially, respondents were asked thirty two items. Each item was scored on a five point Likert Scale ranging from ‘strongly disagree’ to ‘strongly agree’. These items were subjected to exploratory factor analysis with principal components extraction method to identify underlying factors and to reduce the number of items. The factor analysis was conducted as the part of data analysis to prepare the data for further statistical analysis. Factor solution with egenvalue greater than one was retained for further analysis after Varimax rotation method. Consequently, nine meaningful factors emerged and they were named as respect (variance = 9.90%), perceived HEWs’ competency (variance = 9.60%), satisfaction (variance = 8.72%), perceived availability of HEWs at HP (variance = 8.72%), intention to consult HEWs (variance = 8.70%), preference for HP for FP (variance = 7.43%), perceived HEWs’ skill to diagnose community problems (variance = 5.54%), attitude towards home visit (variance = 4.33%) and perceived relevance of HEP (variance = 3.96%). The cumulative percentage of variance explained was 62.82%. During factor analysis, double loaded, negatively loaded and weakly related items (factor loading <0.40) to the emerged factor components were dropped from further analysis, and hence, the number of items was reduced to sixteen.

### Outcome variable

Respondents’ overall satisfaction with health extension program was considered as an outcome variable. User’s satisfaction is considered as one of the desired outcomes of health care and it is directly related with utilization of health services [19]. Three items related to satisfactions, on the five point Likert Scale, were used to assess respondents’ satisfactions with HEP. A median point was considered to label satisfactions with HEP; respondents who scored above the median value were considered as satisfied.

FGD guides were used to collect the qualitative data. The guide mainly covered general issues such as the involvement of both men and women in HEP, availability of HEWs, competency, acceptance and trust, perceived relationships, orientations on static and outreach services, range of services being provided in HEP etc. The questionnaire was translated into Afan Oromo (local language) and retranslated to English to check its consistency. The Afan Oromo version was pre-tested on similar population and used for data collection. The interview was conducted by experienced and trained individuals. The investigators closely supervised the data collection process. Each FGD was conducted and transcribed by MPH holders who were native to the local language. The FGD discussions were tape-recorded besides taking the notes by the FGD facilitators.

### Statistical analysis

The quantitative data were analyzed using SPSS17.0. Descriptive statistics were used to summarize the data. Mean score was computed for each emerged factor component during factor analysis after all items composed of the given factors were summed up. Then, the total score was converted to 100 percent for possible comparisons of the mean scores. For further multiple linear regression analysis, factor score was computed for each factor. To identify factors which significantly predicted satisfactions with HEP, all variables which were significant on Bivariate analysis (p < 0.05) were fitted into regression model. A 95% confidence interval and level of significance less than 0.05 were used to check for association. Beta coefficient was interpreted for statistically significant variables. The data revealed from the FGDs’ were transcribed verbatim into English and analyzed by ATLASti 4.1 qualitative data analysis software. Finally, the qualitative data were narrated and triangulated together with the quantitative findings.

### Ethical consideration

Ethical clearance was obtained from the Ethical Committee of the College of Public Health and Medical Sciences, Jimma University. All respondents were given detailed information about the objective of the study and verbal consent was obtained from each respondent before the interview.

## Results

### Socio-demographic characteristics of the respondents

Three hundred seventy nine respondents were participated in the study producing response rate of 94.0%. Table 1 presents background characteristics of the respondents. Accordingly, 325 (85.8%) of them were married. The mean age of the respondents was 32.8 ± 8.6 years. In terms of religion, 357 (94.2%) of the respondents were Muslims. Nearly all, 375 (98.9%) of the respondents, were farmers and the dominant ethnic group was Oromo, 351 (92.6%).

**Table 1:** Background characteristics of the respondents, Jimma Zone, Dec 2011 (n = 379)

**Socio-demographic characteristics**		**Frequency**	**Percentage**
Married and live together	Yes	325	85.8
	No	54	14.2
Age	15-24	54	14.2
	25-34	156	41.2
	= > 35	169	44.6
Religion	Muslim	357	94.2
	Orthodox	11	2.9
	Protestant	9	2.4
	Others	2	0.5
Ethnicity	Oromo	351	92.6
	Dawuro	11	2.9
	Others*	17	4.5
Occupation	Farmers	375	98.9
	Others**	4	1.1
Is the mother pregnant?	Yes	58	15.3
	No	321	84.7

*Kaffa, Tigire, Ahmara, **merchant, daily laborer,

#### Mothers’ interactions with HEWs

Eighty three (21.9%) of the respondents were accorded the title of model families as adopters of services given by HEWs. Only half, 196 (51.7%), had at least one visit to the HP during the one year prior to the survey with average number of visits of 2.68 ±1.24 times. The FGD discussion also revealed that community members rarely visit HEP mainly due to non-existence of curative services and less availability of HEWs at HP. Of those respondents who visited the HP, the majority, 178 (90.8%), reported having obtained the kind of service they wanted. The remaining percentage visited the HP to seek treatment services and did not get the expected services (Table 2).

**Table 2:** Respondents’ experiences and interactions with HEWs, Jimma, Ethiopia, Dec. 2011

**Variables**	**Response category**	**Frequency**	**%**
Recognized as model family	Yes	83	21.9
	No	296	78.1
Ever visited HP during the last one year	Yes	196	51.7
	No	183	48.3
Frequency of visit during the last one year (mean 2.68 ± 1.24)	= < three times	146	74.5
	= > four times	50	25.5
Time taken to reach at HP on foot (in minutes) (mean 38.80 ± 23.31)	1-30	196	51.7
	31-60	158	41.7
	>61	25	6.6
How do you rate the availability of HEWs on job at health post?	Always	101	26.6
	Occasional	194	51.2
	Rarely	84	22.2
Ever returned home due to HP being closed	Yes	90	23.7
	No	289	76.3
Did HEWs visited your home during the last one year	Yes	271	71.5
	No	108	28.5
How frequently do HEWs conduct outreach services?	Most of the time	112	29.6
	Sometimes/intermittent	245	64.7
	Never	22	5.7
Do they involve your husband?	Yes	177	65.3
	No	94	34.7
HEWs should involve in HEP	Yes	353	93.1
	No	26	6.9
Two HEWs are adequate per Ganda	Yes	164	43.3
	No	215	56.7
Service provided by HEWs are enough	Yes	185	48.8
	No	194	51.2
Where do you think HEWs live?	Health post	116	30.6
	Town	229	60.4
	Others	34	9.0

#### Perceived access to HEWs at HP

Respondents were asked to rate the availability of HEWs at HP. Accordingly, a small percentage of them, i.e., 101(26.6%), rated regular availability of HEWs at HP. Nearly half, 194 (51.2%) of the respondents, observed occasional availability of these workers at HP. In relation to this, 90 (23.7%) of the respondents reported they used to returning home since HP was closed (Table 2). In the qualitative study, also the majority of the participants ascertained that it was hardly possible to get HEWs at HP. According to them, as a result, people suffer from malaria, children discontinued immunization, and women dropped out contraceptive use. On the other hand, they used to face challenges to request services from other centers as their records are available at the HP. They strongly suggested that the HP must be always open.

*“They (HEWs) are available only two days in a week at HP. One may get sick or there may be a woman for delivery. If you go there, you cannot find them. Why does this happen? Always, the health post must be open. The government should either assign more extension workers or let one of them stay at health post.”* (A 34 years old woman)

#### Perceived extent of outreach services

Two hundred seventy one (71.5%) of the respondents stated HEWs were visiting their home during the last one year prior to the survey. Only 177 (65.3%) of the respondents responded that their husbands participated in the discussion about HEP during home visit by HEWs. Respondents were asked to rate how frequently HEWs conducted outreach services. Despite the higher time devoted for home visit, the majority, 245 (64.7%), of the respondents rated that the visit as ‘intermittent’ (Table 2). FGDs participants’ opinions also supported this finding except in one of the districts. For instance, a discussant said:

*“No, I have not seen any HEW at my village. Why should we lie? I have not seen them except that they come to our village for polio vaccination.”* (A 32 years old woman)

#### Respondents’ views on health extension services

The majority of the respondents, 353 (93.1%), supported the involvement of female workers in HEP (Table 2). However, most FGD participants basically did not support the idea that HEP shall be run only by female workers. Their belief is that the involvement of both men and women are vital for the success of HEP. Female workers were preferred for the premises of degree of closeness, easier disclosure of personal problems and as a matter of cultural norms. Associated with cultural norms and biological factors, the fact that mothers are ready to share their personal issues to females than to males. For instance, a discussant said:

*“Because they easily share and understand our problem. If they were males, we would be ashamed to tell them about our secret, about our internal problem (biological). But, we could sincerely share our problems with females and they look into it and give us a piece of advice.” *(A 28 years old woman)

Still, males involvements in HEP was recommended for various reasons. Respondents believed that men are more professional in handling technical issues; have the capacity to withstand challenging work conditions and are right decision makers for immediate action during health hazards. The argument was that females are fearful and subjected to ill-decision. Moreover, it was believed that males are physically and biologically strong to work in risky conditions; are better in improving the availability of drugs and other supplies at HP as they can exert a strong pressure on higher concerned bodies.

A 30 years old woman said:

“In fact, it is difficult for HEWs to go home and assist delivery at night because the environment is not convenient to walk at night; there is no suitable road, it is dark. In such a case, males are more appropriate than females. By nature, men are more active and stronger than women. They can manage these challenges. Males have more acceptance than females. People accept the advice given by males than the advice given by females.”

However, 164 (43.3%) disagreed that two HEWs are adequate per Ganda to carry out health extension services. Added to this, nearly half, i.e., 185 (48.8%), of the respondents did not agree with the range of services being provided by HEWs (Table 2). They felt that treatment services such as eye disease, TB, diarrhea and disease of internal organs ought to be addressed in HEP. Qualitative finding also revealed consistent result. In fact, most discussants highly criticized HEP for absence of curative services and argued that HEWs are not able to handle many of the problems they encounter, specially the curative services.

#### Interpersonal relationship

The study revealed that three-fourth, 286 (75.5%), of the respondents perceived that they had positive interpersonal relationship with HEWs. Added to this most, 316 (83.4%), of them knew HEWs in person. Similarly, most of the respondents, 329 (86.8%), preferred to receive health related information or advice from HEWs. The remaining percentage (14.2%) preferred other individuals such as community volunteers and traditional healers. However, despite good interpersonal relationship, many discussants reported that HEWs had less acceptance and less trusted.

#### Exposure to HEP packages

Table 3 presents respondents’ exposure to HEP packages. Accordingly, 289 (78.6%) of the respondents had received health information from HEWs. The health information focused on multiple topics. In fact, they had better exposure to information on personal hygiene and environmental sanitation compared to others in HEP packages. For instance, 95.6%, 94.0% and 92.6% of the respondents received information on housing hygiene/condition, personal hygiene and environmental hygiene respectively. On the other hand, the proportions of respondents who were exposed to communicable diseases package (eg. Tuberculosis) appeared to be lower (Table 3). A similar pattern was also observed in FGD discussions. Most discussants frequently mentioned that they received information on how to keep personal hygiene, keeping rooms and surrounding clean and how to use toilet than other issues. For instance, a participant said:

*“…. They advice people about constructing toilet; clean their house, discourage open defecation, clean bed rooms and keep their surroundings clean. They also teach about waste management; like separation of liquid waste from solid waste*.” (A 28 years old woman)

**Table 3:** Respondents’ exposure to HEP packages, Jimma, Ethiopia, Dec 2011

**Characteristics**		**Frequency**	**%**
Ever received health information from HEWs ?	Yes	298	78.6
	No	81	21.4
Exposure to different health issues (n = 379)	Nutrition	213	72.9
	FP	263	88.6
	ANC	225	75.8
	Immunization	262	88.2
	Delivery	225	76.0
	Breastfeeding	207	69.7
	Housing hygiene/condition	285	95.6
	Personal hygiene	280	94.0
	Environmental hygiene	276	92.6
	HIV/AIDS	206	70.5
	Malaria	258	87.5
	TB	188	64.4

#### Descriptive statistics for emerged factors

Table 4 contains descriptive statistics for each factor emerged during exploratory factor analysis. A close look into these factors has shown that respect, competency, availability and perceived HEWs’ skill to diagnose community problems were related to HEWs. These four factors together explained 28.6% of the variance. On the other hand, the other three factors were related to mothers’ perceptions about health extension services. They included perceived relevance of HEP, attitude towards home visit and preference. The highest mean score was found for home visit (87.93 ± 16.03) and the second highest mean score for perceived relevance of HEP (86.86 ± 12.04). Qualitative finding also revealed consistent results. The majority of FGD discussants basically recognized that HEP meets their needs and the presence of home visit received higher appreciation. On the other hand, lower mean score was observed for perceived HEWs’ skill to diagnose community problems (69.97 ± 25.91). Most FGD participants also did not trust the capacity and skill of HEWs to handle many of the clients’ problems. Similarly, relatively lower mean score was observed for availability dimension (71.31 ± 17.10). A highly consistent finding was also revealed in qualitative part of the study. Most discussants objected to the lower attention given to daily routine health service at HP (25% time budgeting for static services). In addition, they explained the fact that HEWs frequently called for meeting and training, and lived in towns which contributed for absenteeism. Thus, mothers had unpleasant experiences in relation to access to HEWs at HP when needs arise.

**Table 4:** Mean score for emerged factors, Dec 2011

**Factors components**	**Mean ± SD**	**Range of possible score**
Respect	81.29 ± 15.19	20-100
Competency	75.84 ± 11.78	30-100
Satisfaction	82.99 ± 18.16	20-100
Availability	71.31 ± 17.10	30-100
Intention to consult	76.91 ± 15.56	20-100
Preference of private clinic for FP	79.42 ± 25.33	10-100
HEWs’ skill to diagnose community problems	69.97 ± 25.91	10-100
Home visit	87.93 ± 16.03	10-100
Perceived relevance of HEP	86.86 ± 12.04	10-100

#### Descriptive statistics for satisfaction

In this study, the overall mean of satisfaction score was found to be 83. 0 with standarviation (SD) of 18.2 (range of possible score 20–100) (Table 4). The median point of the data was 86.7 and 69.6% of the respondents scored above the median value indicating satisfaction with HEP delivered by HEWs.

### Predictors of satisfaction with HEP

Table 5 contains regression estimates for variables significantly associated with satisfaction as identified through bivariate analysis. Most of these variables remained significant except home visit and perceived relevance of HEP. Hence, mothers’ satisfaction with HEP was mostly explained by age of the mothers, perceived HEWs’ skill to diagnose community problems, perceived respect, involvement of husband in the program and being titled as model family. Two of these decisive determinant factors were related to HEWs’ professional and interpersonal skills; namely perceived respect and skill to diagnose community problems. Perceived HEWs’ skill, respect and being titled as a model family were best predictors of satisfaction. For instance, a unit increase in perceived respect score would increase the level of satisfaction by an average of 29% (95% CI: 14%–45%, p = 0.001). Similarly, being regarded as a model family increases satisfaction by an average of 15.52 (95% CI: 10.79–20.25, p = 0.001).

**Table 5:** Regression estimates for predictors of satisfaction with health extension service, Dec 2011

**Variables**	**Unstandardized β coefficients**	**Sig.**	**950% CI β**
Age	0.24	0.046	0.01-0.47
HEWs’ skill to diagnose community problems	0.17	0.001	0.09-0.25
Perceived respect	0.29	0.001	0.14-0.45
Perceived relevance of HEP	0.01	0.966	-0.17-0.18
Home visit	-2.06	0.731	-13.85-9.73
Involvement of husband (Yes/No*)	5.15	0.023	0.72-9.58
Model family (Yes/No*)	15.52	0.001	10.79-20.25

*reference category.

## Discussion

The study revealed that only one-fourth of the households were graduated as model family for being adopters of services given by HEWs. This leaves a huge gap since all households were expected to be trained and graduated during the first three years of the program implementation. Previous studies also documented that the overall trend in the graduation of model families is far behind the expectation [3,5,6,10,20]. Literatures documented several reasons regarding low progress in graduating model families such as being overloaded with activities (HEWs), less involvement of community volunteers in the program, lack of incentives for community volunteers, less acceptance of and closed attitudes on the part of the community, uncomfortable working condition and living environment for HEWs, lack of commitment from HEWs, limited comprehensive and supportive supervision, shortage of supplies, poor transportation and communication facilities, lack of access to reference materials and other resources [9-15,20]. However, nearly three-fourth of the respondents received information on some health extension packages during one year before the survey. This finding is found to be better compared to earlier reports [13,16]. Better exposures to information observed for hygiene and environmental sanitation packages. However, consistent with some earlier reports [12,16], community exposure to family health and communicable diseases control package was lower. This might be due to the higher attention laid on the outreach services as program expectations.

HEWs are required to spend 75% of their time conducting outreach activities by going from house-to- house while the remaining 25% at the HP [5] leaving less time for static services. The current finding is consistent with the expectations of the program as more than half of the respondents stated that HEWs were infrequently available at HPs. However, the scenario of 25–75% was a contentious agenda among the FGD participants. It is strongly criticized as it gave less attention to routine daily health services being rendered at HP. This is because, on most of the working days, the HP is closed as both of the HEWs are required to go out for outreach activities. Earlier study also reported similar findings [10]. It was one of the unpleasant experiences mothers had as they could not access to HEWs when they need them, especially for time- sensitive cases such as family planning, illness, immunization and emergency conditions. Consequently, for a large number of respondents, it was not uncommon to return home without getting the service they wanted because of the closure of HPs. Another reflection of this reality was the fact that nearly three-fourth of the respondents preferred private clinics to HPs for family planning service. On the other hand, inconsistent with the expectation, most respondents did not agree with the claim that HEWs were spending most of their time on conducting outreach activities. The qualitative finding also revealed similar experiences: most discussants argued that they were not sure where HEWs spent most of their time. Thus, it is believed that the 25–75% scenario might facilitate absenteeism as it is easier to attribute reasons of absence from work either to static or outreach services. On the top of that, some literature reported that in most cases, HEWs live in uncomfortable environment [12] which may also contribute for absenteeism from work.

The involvement of female HEWs alone in HEP received higher attention, especially among FGD participants. The majority of the participants did not support that HEP shall be run only by female workers. It is believed that active involvement of both females and males is a necessary condition for HEP success. The involvement of female HEWs in the program was preferred on the grounds of degree of closeness, easier disclosure of personal problems and cultural norms. This might reflect the fact that most mothers tend to have better relationship with HEWs. However, the extent of reported relationship was lower compared to an earlier report [16] though higher proportions of respondents knew HEWs by their names as found out in this study.

On the other hand, males’ involvement in HEP was recommended for various reasons such as being more professional/expert and competent, ascribed status in the community and capacity to withstand challenging work conditions. Such preferences might be associated with gender roles and deep rooted cultural beliefs that portray men as more competent, active and brilliant than women. In fact, such beliefs would have a negative impact on the acceptance of HEWs as it was revealed in qualitative part of the study. Similar finding was reported in one previous study [20].

Though, curative health service is not part of HEP packages [5], the current study revealed higher unmeet demands for curative health services. Consistent with some earlier reports [10-12,21], HEP was highly criticized in that it does not encompass curative health services and HEWs cannot deal with many of the health problems the community encounters. Although HEWs administer anti-malaria drugs, several participants complained that they were not given the drugs. Previous studies have also reported problems of anti-malaria drug supply at HP level [10,12]. In addition, one study reported that, in some cases, HEWs are not competent enough to use the anti-malaria drugs even when these drugs are available [12]. On the other hand, there might be over expectations regarding treatment of malaria as HEWs are providing only artemether/lumefantrine drug.

HEP was acknowledged though it has some perceived drawbacks. For instance, the existence of home visit was highly appreciated. Earlier studies also documented similar findings [9,10,13,15,16]. Nevertheless, there were concerns related to programmatic issues such as 25–75% scenario, absence of curative services and lower competency and skill of the HEWs. Limited access to information, resources and reference materials may be associated with the lower competency of HEWs as documented in earlier studies [10-12,14]. Despite these concerns, the level of satisfaction was moderately high which was also documented in earlier researches [9,16]. Finally, age, perceived HEWs’ skill to diagnose community problems, perceived respect, involvement of husband in HEP and being model families were significantly predicted respondents’ satisfactions with health extension service provisions. Older mothers tend to be more satisfied with HEP service. This might be due to difference of expectations between young and older women. The involvement of husbands, during home based health education also plays an important role to boost the level of satisfaction. This might be because the husband is a key decision makers in household matters in Ethiopia. Similarly, being a model family was associated with higher satisfaction implying that families tend to appreciate and give recognition to HEP as they fully pass through all packages of HEP. Some earlier studies also reported that respect and politeness, background characteristics of respondents such as education and age, perceived competency, interpersonal communications and information sharing were powerful predictors of satisfactions with health service [22-28]. However, it is difficult to compare the finding of the current study with earlier reports as earlier reports were based on populations who seek medical care and treatments which is totally incomparable with the service being provided at HP and community level. Nevertheless, there are some determinants of satisfaction which are also common in both settings such as interpersonal relationship, respect and perceived competency of providers.

### Limitations of the study

It must be noted that the finding of this study represent only community perceptions. We did not study the reflection of HEWs and other stakeholders. In addition, the study did not cover large geographic areas which affect generalization of the finding. HEP is new to Ethiopian health care delivery system which limits the comparisons of the current findings with earlier studies on satisfactions with health services.

## Conclusions

Despite these limitations, the following conclusions would be drawn from the current study. Respondents’ perceived that HEP is highly relevant to improve community’s health status. The implementation of home visit received higher recognition. On the other hand, the participation of men in HEP was perceived to be vital for the success of the program though the involvement of women is still believed to be indispensable. This calls for a reconsideration of the program for possible men involvement. However, HEP was criticized primarily because of absence of curative services and the 25–75% scenario. The scenario affected community access to HEWs when need arise. In addition, the skill and competency of HEWs to handle many of the community problems was less trusted calling for the need to advance HEWs competency and skill. Despite these concerns, higher satisfaction and favorable interpersonal relationship were reported. Age of respondents’, perceived HEWs’ skill, perceived HEWs’ respect, involvement of husband in discussion during home visit, and being titled as model family were best predictors of satisfactions with health extension service. The study implies the need for reconsideration of programmatic issues that affects the delivery of HEP including, the scenario of 25–75% and inclusion of basic curative health services. HEWs are required to establish good rapport with community. Further studies are required to investigate the effect of the 25–75% scenario and men involvement in HEP.

## Competing interests

The authors declare that they have no competing interests.

## Authors’ contributions

ZB conceived the study. ZB, YK, AG and MG were involved in the design, field work, data analysis and interpretation, report writing and manuscript preparation. In addition, ZB drafted the manuscript. All authors reviewed, read and approved the final version of the manuscript.

## Pre-publication history

The pre-publication history for this paper can be accessed here:

http://www.biomedcentral.com/1472-6963/13/74/prepub
